# Nuclear Proteomics to Understand the Promotive Effect of Plant-Derived Smoke Solution on Wheat Under Salt Stress

**DOI:** 10.3390/proteomes14020031

**Published:** 2026-06-15

**Authors:** Sheikh Shohag, Hisateru Yamaguchi, Keisuke Hitachi, Kunihiro Tsuchida, Shafiq Ur Rehman, Setsuko Komatsu

**Affiliations:** 1 Department of Applied Sciences and Engineering, Fukui University of Technology, Fukui 910-8505, Japan; 2 Department of Medical Technology, Yokkaichi Nursing and Medical Care University, Yokkaichi 512-8045, Japan; h-yamaguchi@y-nm.ac.jp; 3 Center for Medical Science, Fujita Health University, Toyoake 470-1192, Japan; hkeisuke@fujita-hu.ac.jp (K.H.); tsuchida@fujita-hu.ac.jp (K.T.); 4 Department of Biology, University of Haripur, Haripur 22620, Pakistan; 5 Faculty of Environment and Information Sciences, Fukui University of Technology, Fukui 910-8505, Japan

**Keywords:** wheat, salt stress, plant-derived smoke solution, nuclear proteomics

## Abstract

Background: Salinity, which hampers wheat growth and development, is one of the major abiotic stresses. Plant-derived smoke (PDS) solution alleviates salt stress and promotes wheat growth and development; however, the underlying molecular mechanisms have not been completely clarified. Methods: In this study, nuclear proteomics was employed to reveal the promotive effect of PDS solution on salt-stressed wheat. Nuclear fractions were isolated from wheat roots, and their purity was confirmed via enrichment of histone H3 and reduction of cytosolic ascorbate peroxidase. Using this nuclear purification technique, label-free nano LC–MS/MS-based nuclear proteomics was performed to identify differentially abundant nuclear proteins in salt-stressed wheat with or without PDS solution treatment. Results: Salt stress decreased histone H2A and DNA polymerase levels, whereas PDS solution treatment of salt-stressed wheat increased levels of histone variants (H2A, H2B, H3, and H4), DNA polymerase, and DNA topoisomerase II. In addition, the PDS solution increased the levels of pre-mRNA cleavage factor Im 25 kDa subunit and RNA helicase in salt-stressed wheat. Immunoblot analysis further validated the increase in histone deacetylase levels triggered by the PDS solution treatment in the salt-stressed wheat. Conclusions: These results suggest that PDS solution alters nuclear proteins in a way that contributes to chromatin remodeling and transcription during salt stress.

## 1. Introduction

Wheat serves as a primary source of nutrition for around 36% of the global population since it is a rich source of food calories and protein, and it is cultivated in 70% of agricultural regions worldwide [1,2]. Earth’s climatic pattern is negatively affected by global warming, which causes extreme weather events that have a direct impact on agricultural productivity [3]. Salinity is a major abiotic stressor that reduces wheat growth and production [4]. Salinity-affected cultivated land predominantly falls in dry and semiarid regions with high evaporation, inadequate precipitation, poor drainage, and salty irrigation water [5]. Salinity hampers photosynthesis, enzyme activity, membrane stability, and nutrient uptake, resulting in oxidative stress, ion toxicity, and decreased crop productivity [6,7]. Wheat yield decreases at salinity levels of 6–8 dS m^−1^ [6]. In wheat, salinity results in an ionic imbalance wherein high Na^+^ concentrations disrupt the intake of nutrients such as K^+^ and Ca^2+^ [8], while high Cl^−^ concentrations disrupt the uptake of nutrients by hindering anion uptake [9]. Salinity initially reduces seed germination and has inhibitory effects on wheat root and shoot parameters; later, it changes growth and reproductive behavior, resulting in severe production losses [6]. In wheat, salt tolerance is a complicated trait governed by numerous genes and involving various biochemical and physiological pathways [10]. Establishing the main traits that maintain salt tolerance and understanding their roles in the response of wheat to salinity can lead to more precise crop improvement techniques that protect both yield and grain quality under changing climatic situations.

Salinity lowers soil water potential, making it difficult for wheat to take up water through its roots, which causes osmotic stress [11]. In response, wheat accumulates osmolytes such as proline, glycine betaine, and sugars, which maintain cell turgor, stabilize proteins and membranes, and protect cellular structures [12]. Antioxidant enzymes like superoxide dismutase, catalase, and peroxidase are activated to prevent oxidative damage since salt stress produces reactive oxygen species (ROS) [13]. The ability of wheat to withstand salt stress is dependent on both transcription factors and signaling pathways [12,13]. WRKY transcription factors improve salt-stress tolerance by activating genes that promote flavonoid synthesis [13]. *TaNAC29* and *TabZIP60* improve the salt-stress tolerance of wheat via the abscisic acid (ABA) pathway [13,14]. These findings suggest that wheat may be able to withstand salt stress by modifying intracellular processes.

Smoke and other fire products have a negative impact on plants, but when they are utilized in solution form and bud into water to create plant-derived smoke (PDS) solution, their role changes to a beneficial one [15]. Smoke from fire acts as a key germination cue in post-fire environments, but fire and smoke can harm beneficial soil organisms, deplete minerals, and cause air pollution [16]. PDS solution enhances seed germination, seedling growth, shoot branching, root formation, and flowering and offers tolerance against abiotic stress [17]. The most prevalent chemical substances found in PDS solution that support plant growth and development are butenolides, cyanohydrins, and nitrates. Karrikins from the butenolide chemical family play a major role in protecting plants from various abiotic stresses at low concentrations by improving the breakdown of seed dormancy and indicating vigor by interacting with phytohormones and transcription factors [15,17,18]. PDS solution has been found to protect wheat [19] and rice [20] from salt stress, soybean [21] from flood stress, and grass peas [22] from drought stress. These reports indicate that PDS solution has a promotive effect on plant growth under abiotic stresses.

Flavonoid-biosynthesis-related metabolites are the key bioactive compounds in PDS solution, promoting wheat growth under salt stress by modulating energy metabolism and leveraging the ubiquitin–proteasome system [19]. Moreover, PDS solution alleviates salt stress in wheat by modulating stress-responsive gene expression, leading to improved seed germination and seedling growth [23]. Wheat productivity is declining as a result of increased salt stress driven by climate change [5]. Taken together, this information suggests that PDS solution functions as a potential biostimulant for improving salt tolerance in wheat seedlings through stress-regulatory gene expression and energy metabolism/protein degradation. PDS solution has practical agricultural relevance because salinity severely reduces wheat yields. However, the role of nuclear protein regulation, which is central to transcriptional control and stress signal integration, remains largely unexplored. Salt stress causes major changes inside the nucleus via transcriptional reprograming [24,25], RNA processing [26], and alteration of chromatin-associated proteins [27], which collectively regulate stress-responsive gene expression. Therefore, the current study explores nuclear proteomics to investigate the promotive effects of PDS solution on salt-stressed wheat. To provide mechanistic insights about nuclear protein alterations caused by PDS solution in wheat under salt-stress conditions, a nuclear purification method was developed and applied to wheat roots. Based on morphological analysis, nuclear proteins were analyzed using liquid chromatography (LC) and mass spectrometry (MS)/MS. To validate the nuclear proteomic result, immunoblot analysis was performed.

## 2. Materials and Methods

### 2.1. Plant Material and Treatment

PDS solution was prepared using the procedure presented in [19]. In brief, semi-dried *Cymbopogon jwarancusa* was smoldered in an airtight furnace, and the smoke generated was bubbled through distilled water to obtain a concentrated PDS solution. In a seedling case with 300 mL of silica sand, the seeds of wheat (*Triticum aestivum* L. cultivar Nourin 61) (Nippon Seed Center, Matsumoto, Japan) were sown either with or without using 2000 ppm of PDS solution, a concentration that enhances salt tolerance in wheat [19]. Wheat seedlings were nurtured in a growth chamber at 25 °C with 70% relative humidity and white fluorescent light (12 h of light at 200 µmol m^−2^ s^−1^ and a 12 h dark photoperiod). Three days after being sown, untreated seedlings were divided into control or 200 mM NaCl treatment groups, while PDS-solution-pretreated seedlings were either re-treated with PDS solution or exposed to 200 mM NaCl in PDS solution for 2 days. The weight and length of the leaves and roots were measured 5 days after sowing. For each experiment, 3 separate biological replicates were employed. In each experiment, 20 seeds were sown for each replication of each treatment. Ten seedlings from each replication of each treatment were harvested for the morphological experiment. To create biological replicas, the seeds were sown on different days.

### 2.2. Isolation of Nuclear Fractions

Nuclei were isolated from wheat roots according to the instructions of the manufacturer of the CelLytic PN Isolation/Extraction Kit (Sigma-Aldrich, St. Louis, MO, USA), with some modifications. Every step of the purification process was carried out on ice. A portion (2.0 g) of sample was ground and homogenized using nucleus isolation buffer. The homogenates were then filtered through filtered mesh. The filtrates were centrifuged for 10 min at 1260× *g* for 10 min at 4 °C. The pellet obtained using a nuclear separation buffer containing a protease inhibitor cocktail was resuspended, and Triton was added. The nucleus-containing solution was layered on the top of 2.3 M sucrose cushions. Following centrifugation at 12,000× *g* for 10 min at 4 °C, the middle layer was obtained using a Pasteur pipette in nucleus isolation buffer containing protease inhibitor cocktail. The resultant solution was centrifuged at 12,000× *g* for 5 min at 4 °C, and supernatant was removed. The purified nuclear proteins were vortexed with extraction buffer.

### 2.3. Measurement of Protein Concentration

Protein standards and samples were incubated with XL-Bradford protein assay reagent (Integrale, Tokushima, Japan), and absorbance was measured at 595 nm using a spectrophotometer. A standard curve was prepared using bovine-serum albumin with concentrations ranging from 0 to 2 mg/mL. Protein concentration of the samples was calculated from the bovine-serum albumin standard curve.

### 2.4. Immunoblot Analysis

Laemmli sample buffer (Bio-Rad, Hercules, CA, USA), which contained 62.5 mM Tris-HCl (pH 6.8), 1% SDS, 10% glycerol, and 0.005% bromophenol blue, along with 50 mM dithiothreitol, was added to protein extracts. Proteins (10 µg) were electrophoretically separated on 10% SDS–polyacrylamide gel and then transferred using a semidry transfer blotter onto a polyvinylidene difluoride (PVDF) membrane. Bullet Blocking One reagent (Nacalai Tesque, Kyoto, Japan) was employed to block the blotted PVDF membrane for 5 min. Following blocking, the PVDF membrane was cross-reacted with primary antibodies for 30 min. As primary antibodies, anti-histone H3 (GeneTex, Irvine, CA, USA), anti-calnexin [28], and anti-ascorbate peroxidase [29] antibodies were used. The secondary antibody applied was anti-rabbit IgG along with horseradish peroxidase (Bio-Rad). After 30 min of incubation, signals were detected with a solution of 3,3′,5,5′-tetramethylbenzidine (Nacalai Tesque). Image J software (version 1.54, National Institutes of Health, Bethesda, MD, USA) was used to compute the integrated densities of the bands.

### 2.5. Enrichment, Reduction, Alkylation, and Digestion of Proteins

Previously reported approaches were used to enrich, reduce, alkylate, and digest proteins [21]. Briefly, quantified proteins (100 µg) were adjusted to a final volume of 100 µL. Methanol, chloroform, and water were added in the following order: 400 µL, 100 µL, and 300 µL, respectively. Following phase separation via centrifugation at 16,000× *g* for 10 min, the upper phase was discarded, and 300 µL of methanol was added to the lower phase and then centrifuged at 16,000× *g* for 10 min. After discarding the supernatant, the pellet was resuspended in 50 mM ammonium bicarbonate. Proteins were reduced for 30 min at 56 °C using 50 mM dithiothreitol and alkylated for 30 min at 37 °C in the dark using 50 mM iodoacetamide. Following incubation, proteins were digested for 16 h at 37 °C with trypsin and lysyl endopeptidase (Wako, Osaka, Japan) in a 1:100 enzyme/protein ratio. Peptides were desalted with MonoSpin C18 Column (GL Sciences, Tokyo, Japan) and acidified with 1% trifluoroacetic acid.

### 2.6. Identification of Proteins Using Nano LC-MS/MS

A previous study outlined the nanoLC conditions (EASY-nLC 1000—Thermo Fisher Scientific, San Jose, CA, USA) and MS acquisition (Orbitrap Fusion ETD MS—Thermo Fisher Scientific) [21]. The peptides were loaded onto an LC system equipped with a trap column (Acclaim PepMap 100 C18 LC column, 3 µm, 75 µm ID × 20 mm—Thermo Fisher Scientific), equilibrated with 0.1% formic acid, and eluted with a linear acetonitrile gradient (0–35%) in 0.1% formic acid at a flow rate of 300 nL min^−1^. Eluted peptides were transferred to an EASY-Spray C18 analytical column (3 µm, 75 µm ID × 150 mm—Thermo Fisher Scientific) for separation, using a spray voltage of 2 kV and an ion transfer tube temperature of 275 °C. The peptide ions were detected using MS in data-dependent acquisition mode with the installed Xcalibur software (version 4.7.69.37—Thermo Fisher Scientific). Full-scan mass spectra were acquired in the MS over 375–1500 *m*/*z* with a resolution of 120,000. The most potent precursor ions were picked for collision-induced fragmentation in the linear ion trap with a normalized collision energy of 35%. In order to stop the repeated selection of peptides, dynamic exclusion was used within 60 s.

### 2.7. Analysis of MS Data

MASCOT (version 2.6.2; Matrix Science, London, UK) and SEQUEST HT search algorithms were used against the UniprotKB *Triticum aestivum* (sp_tr_incl_isoforms_TaxID=4565_and_subtaxonomies; version 2025-10-15) in Proteome Discoverer 2.4 (version 2.4.1.15—Thermo Fisher Scientific). The workflow was outlined in previous research [21]. Spectrum files RC, spectrum selector, MASCOT, SEQUEST HT search nodes, percolator, ptmRS, and minor feature detector nodes were included in both algorithms. Methionine oxidation was considered a variable modification, while cysteine carbamidomethylation was regarded as a fixed modification. The MS and MS/MS mass tolerances were set to 10 ppm and 0.6 Da, respectively. Trypsin was designated as a protease, and a maximum of 2 missed cleavages was allowed. The false-discovery rate was calculated using target–decoy database searches, and it was set to 1% for peptide identification.

### 2.8. Differential Analysis of Proteins Using MS Data

Label-free quantification was performed using Proteome Discoverer 2.4 (Thermo Fisher Scientific) with the precursor ions quantifier node. No additional sample-wise normalization was applied at this stage. Principal component analysis was also conducted in Proteome Discoverer. Downstream statistical analyses were performed using Perseus (version 1.6.15.0) [30]. Protein abundances were log_2_-transformed. To reduce systematic global differences in signal intensity across samples, global median centering was applied by subtracting the median value of each sample, assuming that the majority of proteins were not differentially expressed. Each group consisted of three biological replicates, and proteins were required to have at least three valid values per group. Missing values were imputed from a normal distribution (width = 0.3, down shift = 1.8). Statistical significance was assessed using Student’s *t*-test. Functional annotation of differentially abundant proteins was performed using the gene-ontology database (http://www.geneontology.org/; accessed 26 January 2026) and categorized into cellular components, biological processes, and molecular functions.

### 2.9. Crude Protein Extraction and Immunoblot Analysis

Using a mortar and pestle on ice, a portion (500 mg) of samples was ground in 500 µL of lysis buffer that contained 50 mM Tris-HCl (pH 7.6), 100 mM NaCl, 1% Nonidet-P40, 0.1% SDS, and protease inhibitor (Nacalai Tesque). The suspension was centrifuged twice at 16,000× *g* for 10 min at 4 °C. Protein concentration was determined using the method described earlier in this study. Laemmli sample buffer was added to protein extracts. Immunoblot analysis was performed as described previously in this study. Anti-histone deacetylase antibody (Cosmo Bio, Tokyo, Japan) was used as primary antibody.

### 2.10. Statistical Analysis of Data

The statistical significance of the data between the 2 groups was analyzed via Student’s *t*-test. A *p*-value of less than 0.05 was considered statistically significant.

## 3. Results

### 3.1. Nuclear Purification from Wheat Roots and Purity Evaluation

**Figure 1:**
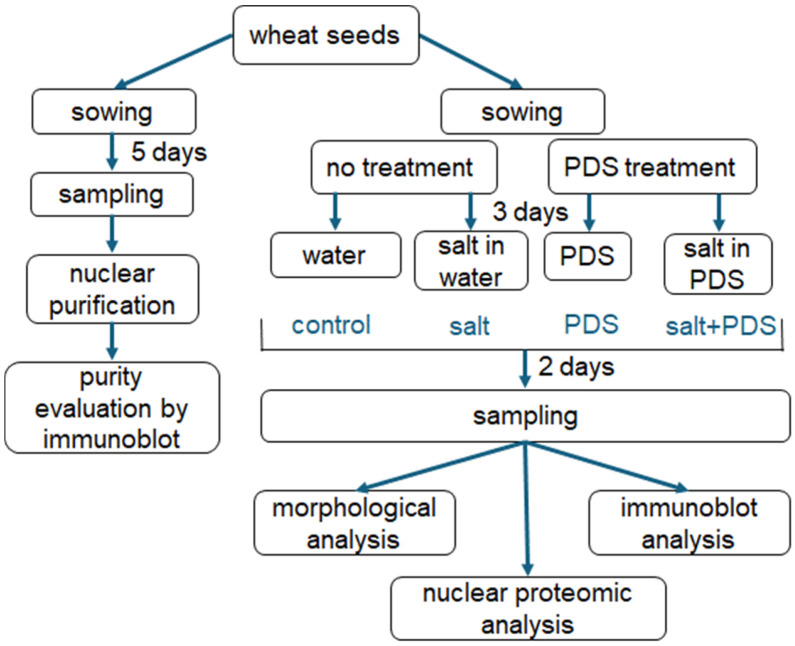
The experimental design for the investigation of the promotive effect of PDS solution on wheat under salt stress. Seeds were sown and treated with or without 2000 ppm of the PDS solution. After 3 days of sowing, wheat was treated with 200 mM NaCl for 2 days. Wheat seedlings were utilized for morphological analysis, nuclear proteomic analysis, and immunoblot analysis. All experiments were performed with 3 independent biological replicates.

**Figure 2:**
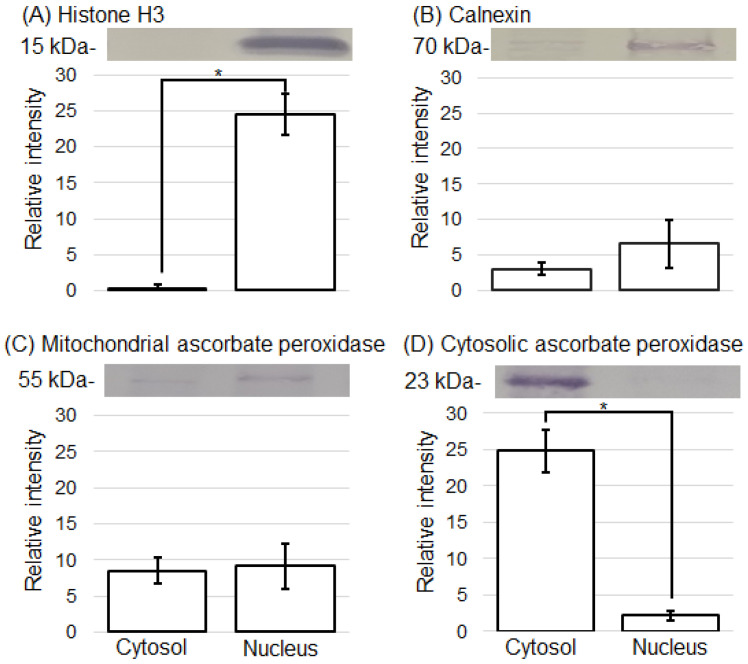
Purity assessment of nuclear fraction from wheat roots. The Coomassie brilliant blue staining pattern was employed as a loading control (Figure S1). As compartment-specific markers, anti-histone H3 (**A**), anti-calnexin (**B**), and anti-ascorbate peroxidase (**C**,**D**) were used. The data represent means ± SDs from 3 independent biological replicates (Figure S1). Statistical comparison between cytosolic and nuclear fractions was performed using a Student’s *t*-test, with asterisks denoting statistically significant differences (* *p* ≤ 0.05).

Nuclear proteomics analysis was performed in order to understand the function of PDS solution in wheat salt-stress tolerance. Before the effects of PDS solution were analyzed, a nuclear purification procedure was optimized. The quality of the nuclear fraction was assessed via immunoblot analysis after nuclear proteins were isolated from the roots of 5-day old wheat seedlings (Figure 1). Coomassie brilliant blue staining was used as a loading control for electrophoresis. The nuclear protein marker was anti-histone H3 antibody. In comparison to the cytosolic fraction, histone H3 was significantly abundant in the nuclear fraction (Figure 2). The purified nuclear fractions were subsequently evaluated for contamination from the endoplasmic reticulum, mitochondrial, and cytosolic fractions. Anti-calnexin, anti-mitochondrial ascorbate peroxidase, and anti-cytosolic ascorbate peroxidase antibodies were used as protein markers for the endoplasmic reticulum, mitochondria, and cytoplasm, respectively. Cytosolic ascorbate peroxidase was accumulated in the cytosolic fraction relative to the nuclear fraction, and calnexin or mitochondrial ascorbate peroxidase were negligible in the nuclear fraction (Figure 2). Overall, immunoblot analysis with organelle-specific markers demonstrated that nuclear proteins were enriched in the nuclear fraction, and endoplasmic reticulum and mitochondrial proteins did not contaminate the nuclear fractions. This nuclear purification approach was utilized to explore the subcellular functions of PDS solution in salt-stressed wheat.

### 3.2. Morphological Changes in Wheat Treated with PDS Solution Under Salt Stress

**Figure 3:**
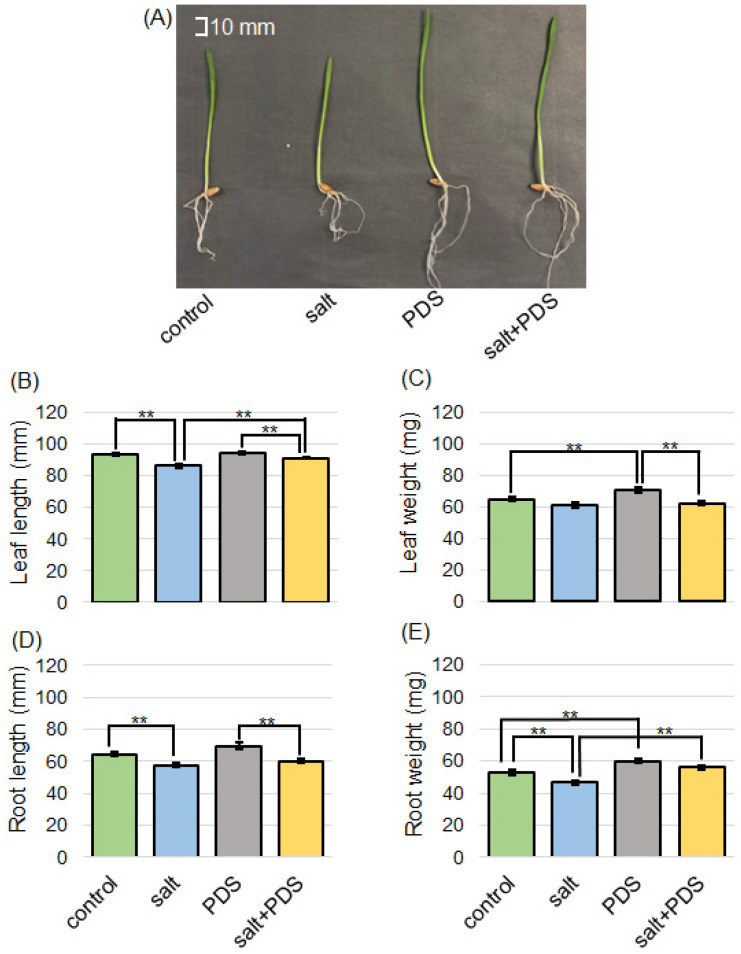
The morphological effects of the PDS solution on wheat under salt stress. Wheat seeds were sown and treated with or without 2000 ppm of the PDS solution. Three-day-old wheat seedlings were treated with or without 200 mM NaCl for 2 days. The bar in the left panel indicates 10 mm in the picture (**A**). As morphological parameters, leaf length (**B**), leaf weight (**C**), root length (**D**), and root weight (**E**) were analyzed 5 days after the seeds were sown. The data are presented as means ± SDs from three independent biological replicates. Student’s *t*-test was utilized to indicate the significant differences between two groups through asterisks (** *p* < 0.01).

The impact of the PDS solution on wheat under salt stress was evaluated using morphological analysis. Leaf length, leaf weight, root length, and root weight were measured as morphological parameters (Figure 3). With the exception of leaf weight, salt stress significantly decreased the leaf length, root length, and root weight of the wheat seedlings. Even though the plants were under salt stress, application of the PDS solution resulted in an increase in leaf length and root weight. PDS solution independently promoted increases in leaf and root weight. Leaf length, leaf weight, and root length were significantly reduced by salt stress in the PDS-solution-pretreated wheat seedlings relative to the group subjected to PDS solution treatment alone, whereas root weight did not change (Figure 3). Wheat roots were employed for nuclear proteomic analysis based on morphological findings.

### 3.3. Identification and Functional Investigation of Nuclear Proteins in Wheat Treated with PDS Solution Under Salt Stress

**Figure 4:**
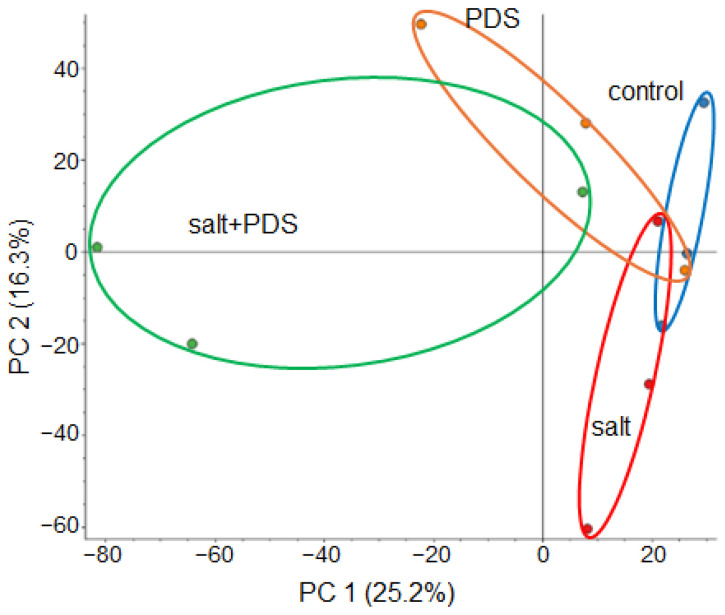
Summary of proteomic results regarding nuclear proteins in wheat roots treated with PDS solution under salt stress. Wheat seeds were sown with or without PDS solution. After 3 days of sowing, wheat seedlings were treated with or without salt stress for 2 days. After purification of nuclei, proteomic analysis was performed, with 3 independent biological replicates for each treatment. Principal component analysis of total proteomic data was performed with 12 samples of nuclear fractions.

Gel-free/label-free proteomic analysis of nuclear proteins was carried out to investigate alterations in the nuclear proteome associated with wheat-root growth under salt stress in response to the utilization of PDS solution. The four different types of treatments, which are the control, salt, PDS, and salt + PDS, were carried out. Nuclear proteins were extracted from 5-day-old wheat roots and analyzed via LC-MS/MS after pretreatment. In total, 6362 proteins were identified. Using principal component analysis, the proteomic data on all 12 samples from the four groups were compared, revealing the diverse protein accumulation patterns resulting from the four kinds of treatment (Figure 4).

**Figure 5:**
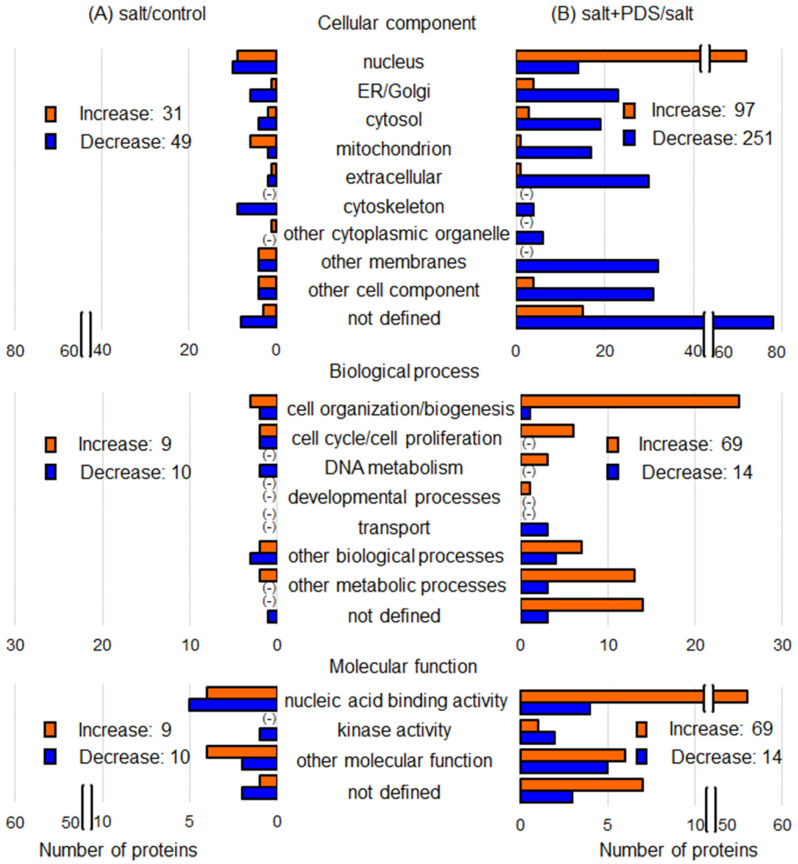
Summary of functional categories of nuclear proteins treated with PDS solution under salt stress. The experimental procedure was the same as that shown in Figure 4. Significantly changed proteins were compared as follows: salt/control (**A**) (Table S1) and salt + PDS/salt (**B**) (Table S2). Functional categories of changed proteins were determined using gene-ontology analysis. Orange and blue columns show proteins whose levels increased and decreased, respectively. “(−)” means that there are no proteins in this category.

**Figure 6:**
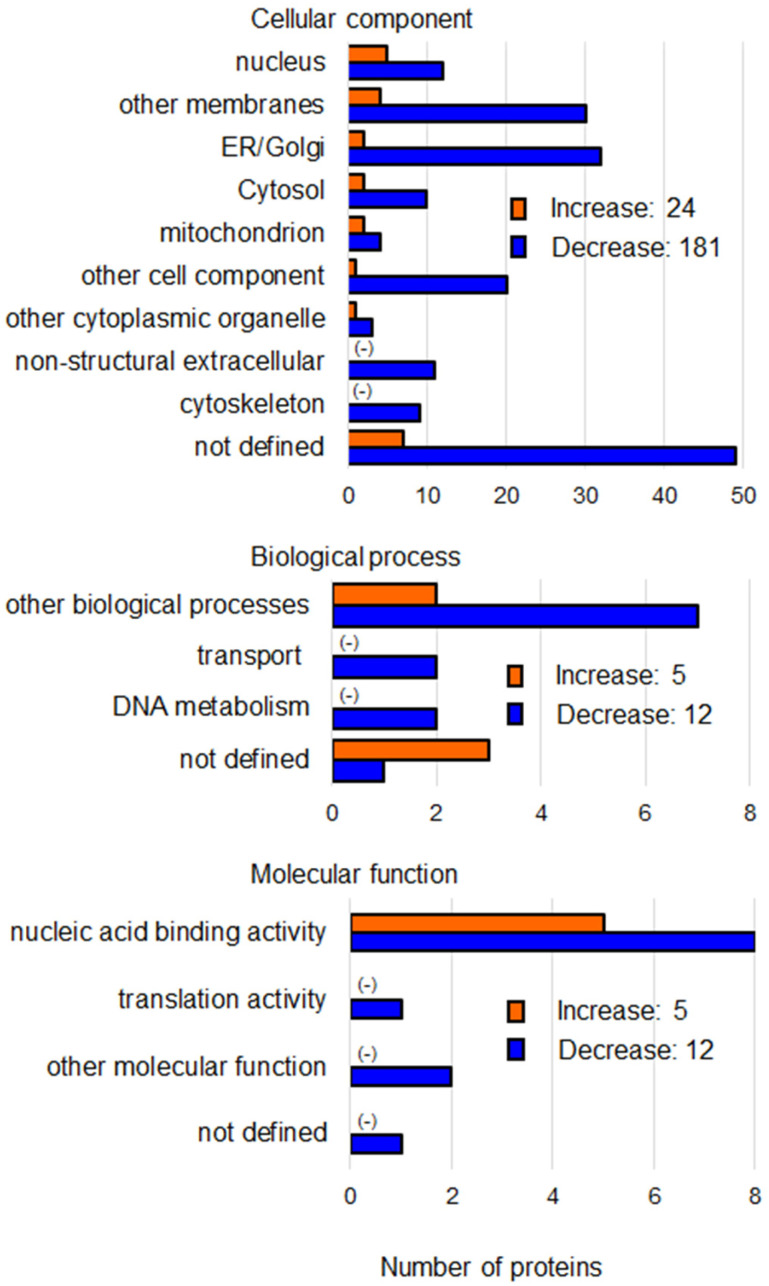
Summary of functional categories of nuclear proteins treated with PDS solution under salt stress relative to PDS solution treatment alone. The experimental procedure was the same as that shown in Figure 4. Significantly changed proteins were compared as follows: salt + PDS/PDS (Table S4). Functional categories of changed proteins were determined using gene-ontology analysis. Orange and blue columns show proteins whose levels increased and decreased, respectively.

The abundance of differentially changed nuclear proteins was evaluated, with the matched peptides ≥ 2 and *p*-values < 0.05. Oppositely regulated nuclear proteins were identified in four comparisons: salt stress compared to the control (Table S1), PDS solution treatment under salt stress compared to salt stress alone (Table S2), PDS solution treatment compared to the control (Table S3), and PDS solution treatment under salt stress compared to PDS solution treatment alone (Table S4). Among 80 proteins in wheat roots under salt stress, compared to the control, the levels of 31 proteins had increased, and the levels of 49 proteins had decreased (Table S1 and Figure 5). However, in contrast to salt stress alone, the abundance of an additional 348 proteins varied in the wheat roots treated with PDS solution under salt stress, with the levels of 97 proteins having increased and those of 251 proteins having decreased (Table S2 and Figure 5). The functional category of identified proteins was obtained using gene-ontology analysis (Figure 5). Altered nuclear proteins were involved in biological processes associated with cell organization/biogenesis and cell cycle/cell proliferation. Kinase activity and nucleic-acid-binding activity were also associated with molecular function. The outcomes of the proteomic study were subsequently examined and validated through immunoblot analysis. The abundance of 205 proteins had differentially changed in the wheat roots subjected to the PDS solution under salt stress relative to the PDS solution treatment alone (Table S4). Among the 205 proteins, the levels of 24 and 181 proteins increased and decreased, respectively. Altered nuclear proteins were associated with nucleic-acid-binding activity in the molecular function category (Figure 6).

### 3.4. Protein Abundance in Histone Deacetylase Modified with PDS Solution Under Salt Stress

**Figure 7:**
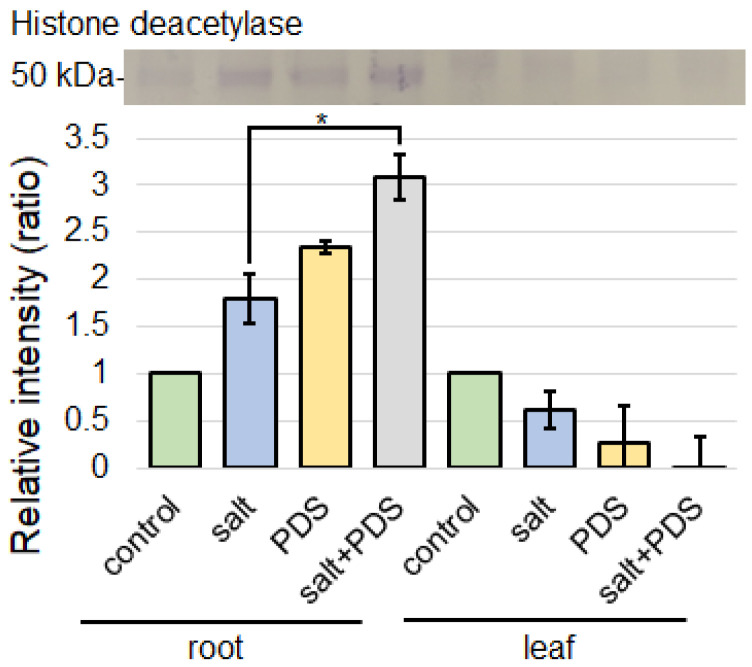
The abundance of histone deacetylase in wheat treated with PDS solution under salt stress. Proteins extracted from wheat roots and leaves were separated using SDS–polyacrylamide gel electrophoresis, transferred onto membranes, and cross-reacted with anti-histone deacetylase antibody. A staining pattern with Coomassie brilliant blue was used as a loading control (Figure S3). The integrated densities of the bands were calculated using ImageJ (version 2.4) software. The data are presented as means ± SDs from 3 independent biological replicates (Figure S4). Student’s *t*-test was used to compare values between two groups. Asterisks indicate significant changes (* *p* ≤ 0.05).

To better evaluate the different treatment-induced changes in nuclear protein accumulation, immunoblot analysis was performed to examine histone deacetylase levels in the nucleus (Figure 7). Proteins extracted from wheat roots and leaves were separated using SDS–polyacrylamide gel electrophoresis and stained with Coomassie brilliant blue, which was used as a loading control (Figure S3). Proteins transferred onto PVDF membranes were cross-reacted with anti-histone deacetylase antibody (Figure S4). The accumulation of histone deacetylase significantly increased in the salt-stressed wheat treated with PDS solution relative to the wheat subjected to salt stress alone.

## 4. Discussion

### 4.1. Development and Optimization of Nuclear Purification in Wheat

The nucleus, a dynamic organelle, carries almost all of the genetic information and changes its proteoforms in response to external stimuli [31]. Numerous cellular processes are managed by nuclear proteins and control intricate regulatory networks [32]. Nuclear proteomics is an effective approach to investigating the mechanisms responsible for plant development and stress adaptation [33]. Nuclear proteomics, which is highly dependent on the purity of the nucleus preparation, helps to identify a specific set of proteins in the nucleus [34]. The establishment of an appropriate nuclear purification technique was an essential step, as the purity of isolated nuclear fractions has a substantial impact on nuclear proteomic accuracy. The nuclear purification procedure effectively enriched nuclear proteins and reduced quantities of non-nuclear proteins in this study (Figure 2). However, in this study, only immunoblot analysis was performed with an organelle-specific antibody to assess the purity of the nucleus. Enzymatic analysis intended to evaluate the purity of the nucleus [35] was not performed, which is one of the limitations of this study. It establishes a solid experimental foundation for exploring nuclear-level adaptation mechanism in wheat treated with PDS solution under salt stress.

### 4.2. Morphological Responses of Wheat to Salt Stress and PDS Solution Treatment

PDS solution is a natural biostimulant, facilitating plant health and endowing plants with the ability to tolerate abiotic stress [15]. Apart from promoting seed germination, PDS solution also increases the length and weight of maize [36] and chickpea [37] seedlings. Soybean growth was enhanced by PDS solution [21,38]. PDS solution has also been observed to protect soybean from flood stress [21] and grass peas from drought stress [22]. PDS solution improves the salt tolerance of soybean [39], wheat [19], and rice [20]. The morphological assessment in the current study (Figure 3) supports the conclusion of an earlier study [19] that PDS solution promotes the growth of salt-stressed wheat. PDS solution enhances soybean root growth through transcriptional promotion with the expression of *RNA polymerase II* [38]. PDS solution oppositely changes proteins categorized in the nucleus in soybean [39] and wheat [19] under salt stress, thus differing from salt stress alone. The morphological recovery in this study suggests that PDS solution may have an impact on molecular regulatory pathways, especially in the nucleus, which regulates the expression of genes under salt stress.

### 4.3. PDS Solution Mediated Modulation of Nuclear Proteins in Salt-Stressed Wheat

In this study, nuclear proteomic analysis was performed to explore the effect of PDS solution on the mechanism controlling salt-stress tolerance in wheat. Nuclear proteomic analysis revealed that histone H2A levels significantly decreased under salt stress (Table S1) but increased in wheat treated with PDS solution under salt stress (Table S2). Histone H2A consists of H2A, H2A.B, H2A.X, and H2A.Z variants, among which the levels of the H2A.Z variant decreased in response to salt stress [40,41]. PDS solution treatment increased histone H2B, H3, and H4 levels in wheat, even under salt stress (Table S2). DNA wraps around core histone proteins such as H2A, H2B, H3, and H4 in eukaryotic cells to create chromatin, which controls gene expression under abiotic stresses such as salt and drought [42]. Histone variants play critical roles in chromatin organization and thereby regulate transcription, chromosome segregation, and DNA damage repair [43]. These findings, combined with the results of our study, suggest that salt stress may affect transcriptional responses and that PDS solution may improve salt-stress tolerance by reshaping key regulatory proteoforms involved in transcription and chromatin organization.

Nuclear proteomic analysis revealed that histone deacetylase levels were significantly increased by PDS solution in the salt-stressed wheat (Table S2). Histone acetylation is a type of reversible post-translational modification [44]. Histone deacetylase is responsible for removing the acetyl group from histones, which causes chromatin condensation and gene silencing [45]. Histone deacetylase 2 (HD2) is a plant-specific histone deacetylase [46]. Salt stress decreases *AtHD2C* expression in *Arabidopsis* [47], while overexpression of *AtHD2C* decreases transcription and increases salt tolerance [46]. In rice, overexpression of *OsHDT701* improves salt tolerance [48]. Based on proteomic analysis, immunoblot was used for confirmation (Figure 7). Although immunodetection is a powerful tool using antibodies [49], the availability of high-quality antibodies for plant proteins is still limited [50]. The immunoblot analysis in this study was performed only with histone deacetylase antibody. Furthermore, transcript-level validation using PCR analysis was not performed in this study. The abundance of histone deacetylase in immunoblots was the same as in the result of the proteomic analysis (Figure 7, Table S2). These results suggest that PDS solution may improve salt-stress tolerance by controlling chromatin modification.

DNA polymerase levels in wheat under salt stress decreased in this nuclear proteomic study, whereas DNA polymerase and DNA topoisomerase II levels increased with the addition of more PDS solution (Tables S1 and S2). DNA polymerases maintain reproduction of genetic information and DNA repair, whereas DNA topoisomerases control topological processes that include the winding of DNA supercoils formed during replication and transcription [51,52]. In rice, DNA polymerase λ activity increases in response to salt and drought stress to facilitate stress tolerance and nuclear DNA damage repair [53]. Plants generate ROS signals in response to environmental stress, and DNA topoisomerases are involved in the integration of these signals [54]. Elevated levels of DNA topoisomerase II improved salt tolerance in tobacco plants with accelerating proline accumulation, wherein proline acted as an osmolyte [55]. The present result, along with previous findings, suggests that treatment with PDS solution enhances genome stability in salt-stressed wheat by promoting the abundance of DNA-replication-related enzymes. This coordinated regulation may facilitate DNA repair, chromatin remodeling, and transcriptional adjustment, thereby contributing to improved tolerance of salt stress.

In salt-stressed wheat, treatment with PDS solution increased levels of pre-mRNA cleavage factor Im 25 kDa subunit (CFIm25) and RNA helicase (Table S2). CFIm is an essential pre-mRNA 3′end-processing factor, and CFIm25 is a part of the CFIm complex [56]. In *Arabidopsis*, *CFIm25* maintained the diversity of the 3′-ends of mRNA [57]. In *Spartina alterniflora*, the expression level of *CFIm25* increased in response to extreme salt stress [58]. RNA helicase is crucial for RNA maturation [59]. *TaDEAD-box57-3B* was up-regulated in wheat and improved tolerance to drought and salt stresses [60]. Overexpression of RNA helicase provided salt tolerance in cotton [61], tobacco [62], and *Arabidopsis* [63]. Increased CFIm25 and RNA helicase levels in PDS-solution-treated salt-stressed wheat might play an important role in the response to salt stresses by adjusting the 3′-end processing of mRNAs and RNA maturation.

Although the present study reveals clear PDS-solution-associated changes in the wheat nuclear proteome under salt stress, the bottom-up LC-MS/MS approach used here has limitations in terms of revealing the true nature of functional proteins. Protein isoforms, combinatorial post-translational modifications, and other proteoform-specific characteristics may not be completely resolved in the bottom-up approach because proteins are enzymatically digested before analysis, and the data represent peptide-based inferences rather than direct characterization of intact proteoforms [64]. While the observed proteomic changes provide biologically meaningful insights into the promotive effect of PDS solution, additional top-down proteomic and targeted validation investigations will be required to describe the whole proteoform and identify specific regulatory mechanisms.

## 5. Conclusions

PDS solution improved wheat growth under salt stress; however, its role has not been completely clarified. To comprehend the role of PDS solution in enhancing salt stress tolerance, a nuclear proteomics analysis of wheat root was performed. The main findings/achievements of this study are as follows: (i) an appropriate nuclear purification method was established, wherein a purified nuclear fraction greatly increased histone H3 levels and reduced cytosolic contamination; (ii) oppositely changed nuclear proteins were associated with cell organization/biogenesis in biological processes and nucleic-acid-binding activity in molecular functions with or without PDS solution under salt stress; and (iii) immunoblot analysis confirmed that histone deacetylase levels increased with the addition of PDS solution in salt-stressed wheat. These findings suggest that PDS solution acts as a natural biostimulant that enhances salt tolerance in wheat seedlings through alteration of nuclear proteoforms, potentially promoting chromatin remodeling and transcription. The utilization of PDS solution may have practical relevance with respect to improving the salt tolerance of wheat. However, additional validation of candidate proteins and functional analyses will be required to clarify the precise regulatory mechanisms underlying PDS-mediated salt tolerance.

## Data Availability

MS data, RAW data, peak lists, and result files of proteomic analysis have been deposited in the ProteomeXchange Consortium [65] via the jPOST [66] partner repository under the data-set identifier PXD074498.

## Supplementary Materials

The following supporting information can be downloaded at https://www.mdpi.com/article/10.3390/proteomes14020031/s1. Table S1. The list of changed nuclear proteins in wheat roots under salt stress compared with the control. Table S2. The list of changed nuclear proteins in wheat roots with PDS solution under salt stress compared with salt stress alone. Table S3. The list of changed nuclear proteins in wheat roots with PDS solution compared with control. Table S4. The list of changed nuclear proteins in wheats root with PDS solution under salt stress compared with PDS solution alone. Figure S1. The Coomassie-brilliant blue staining pattern of proteins used for nuclear purification method development. Figure S2. Blots of the entire membrane with anti-histone H3, anti-calnexin, and anti-ascorbate peroxidase antibodies. Figure S3. The Coomassie brilliant blue staining pattern of proteins used for immunoblot analysis. Figure S4. Blots of the entire membrane with anti-histone deacetylase antibody.

## Abbreviations

The following abbreviations are used in this manuscript:

PDS	plant-derived smoke
ROS	reactive oxygen species
ABA	abscisic acid
LC-MS	liquid chromatography and mass spectrometry
