# Supplementary material for: Nuclear Proteomics to Understand the Promotive Effect of Plant-Derived Smoke Solution on Wheat Under Salt Stress
Source: Proteomes. 2026 Jun 15;14(2):31. doi: 10.3390/proteomes14020031 (PMC13306840; doi:10.3390/proteomes14020031)
Supplement: Supplementary file 1 [file proteomes-14-00031-s001.zip › proteomes-4244056-supplementary Figures.pdf]

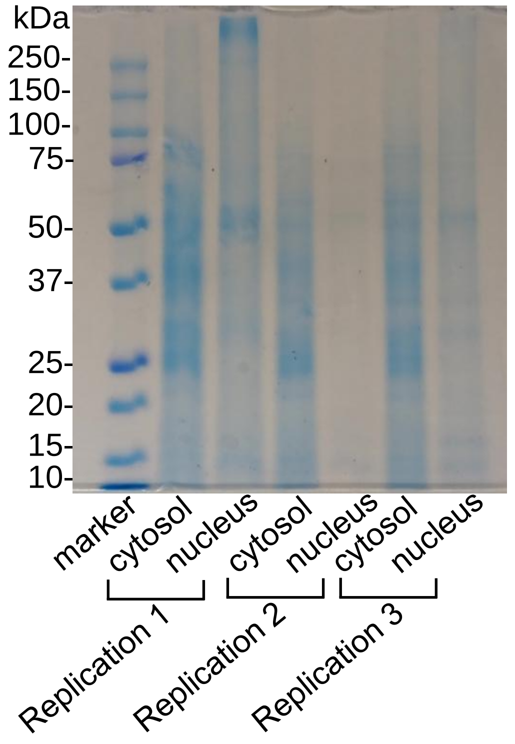

Figure S1. The Coomassie-brilliant blue staining pattern of proteins used for nuclear purification method development. Experiments were performed with biologically triplicates. Quantified proteins (10 μg) from wheat roots were separated by electrophoresis on a 10% SDS-polyacrylamide. Coomassie-brilliant blue staining was used as a loading control.

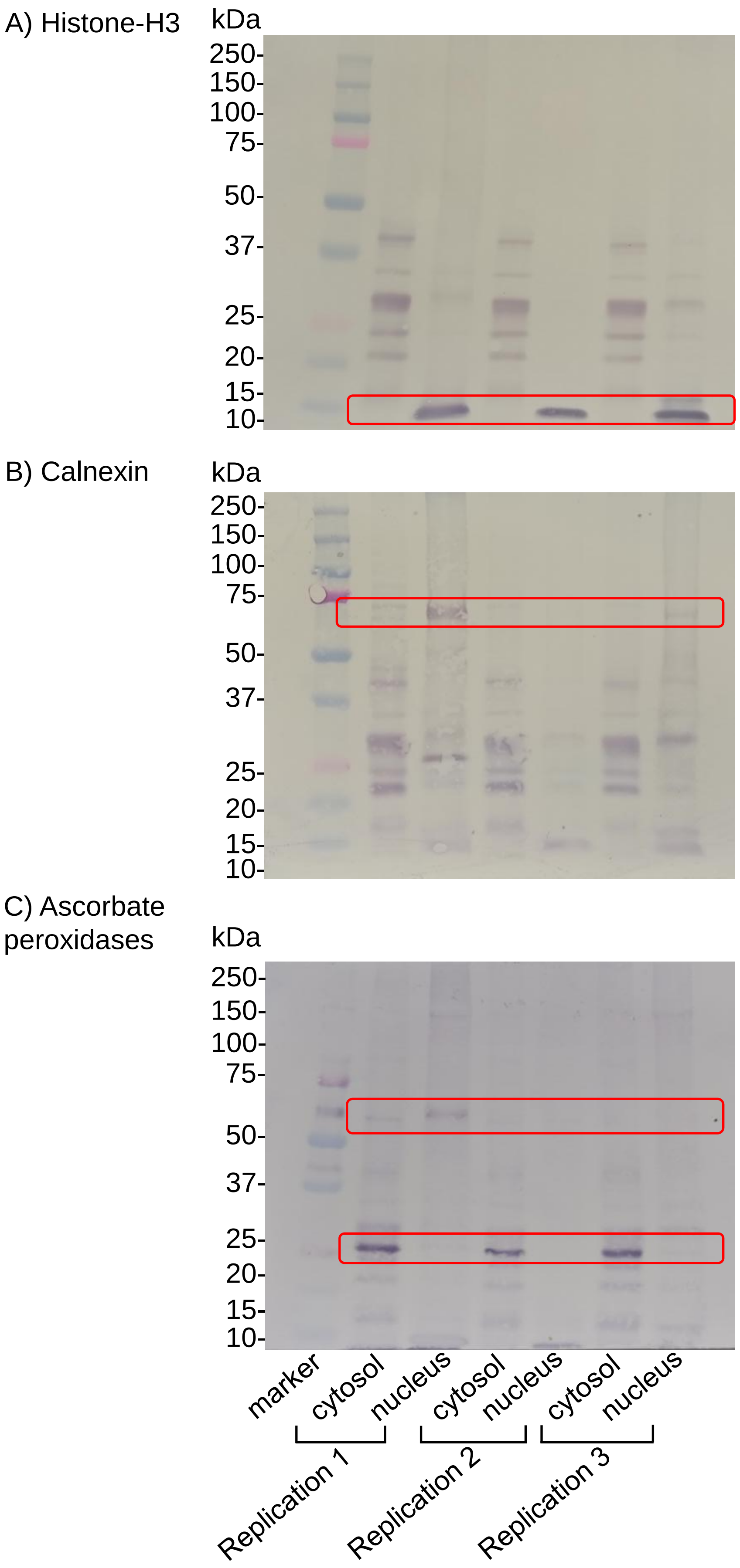

Figure S2. Blots of the entire membrane with anti-histone H3 (A), anti-calnexin (B), and anti-ascorbate peroxidases (C) antibodies, which were used in Figure 2. In Figure 2C, 55 kDa for mitochondrial ascorbate peroxidase and 23 kDa for cytosolic ascorbate peroxidase.

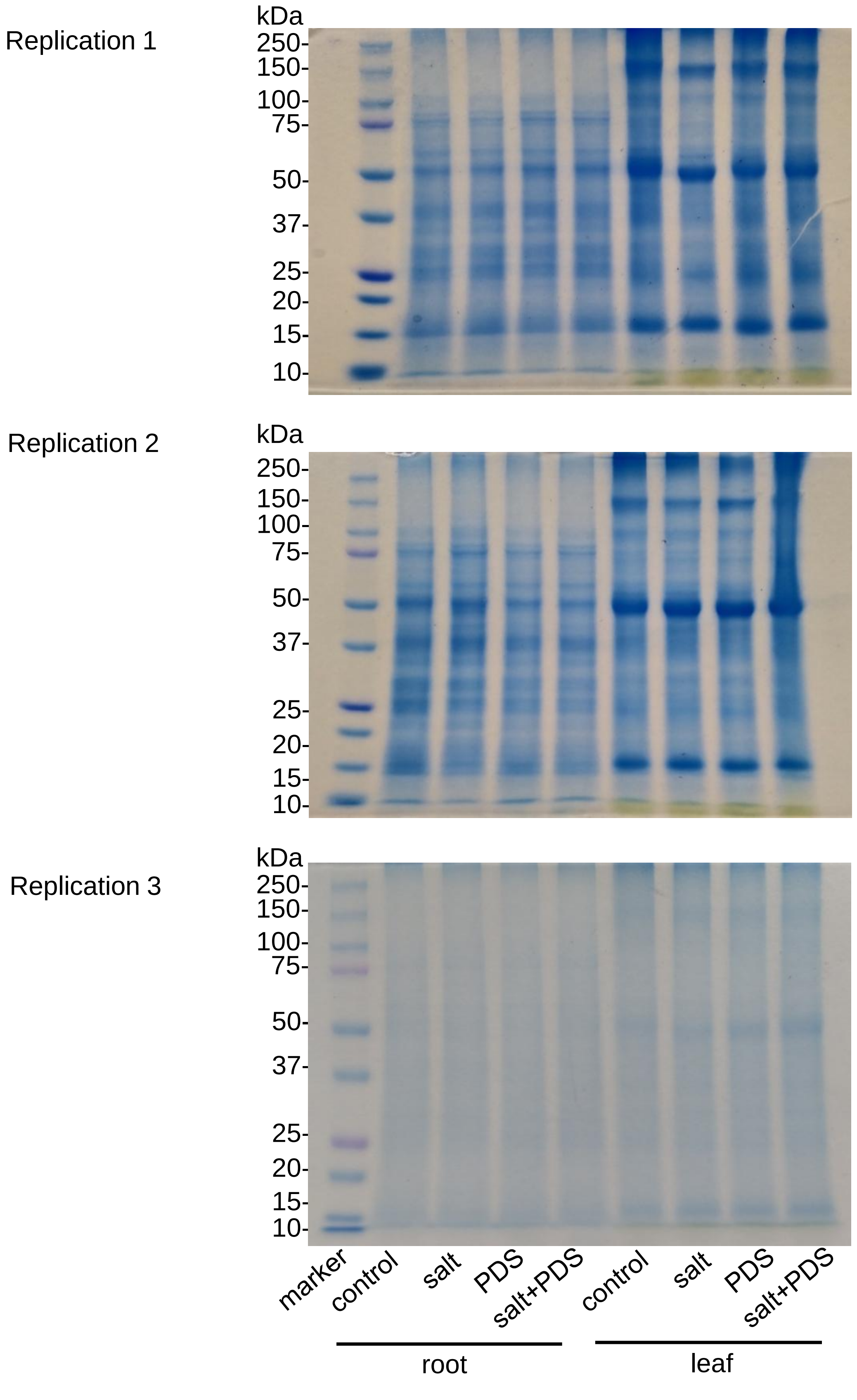

Figure S3. The Coomassie-brilliant blue staining pattern of proteins used for immunoblot analysis. Experiments were performed with biologically triplicates for each treatments. Quantified proteins (10 µg) from roots and leaves were separated by electrophoresis on a 10% SDS-polyacrylamide. Coomassie-brilliant blue staining was used as a loading control.

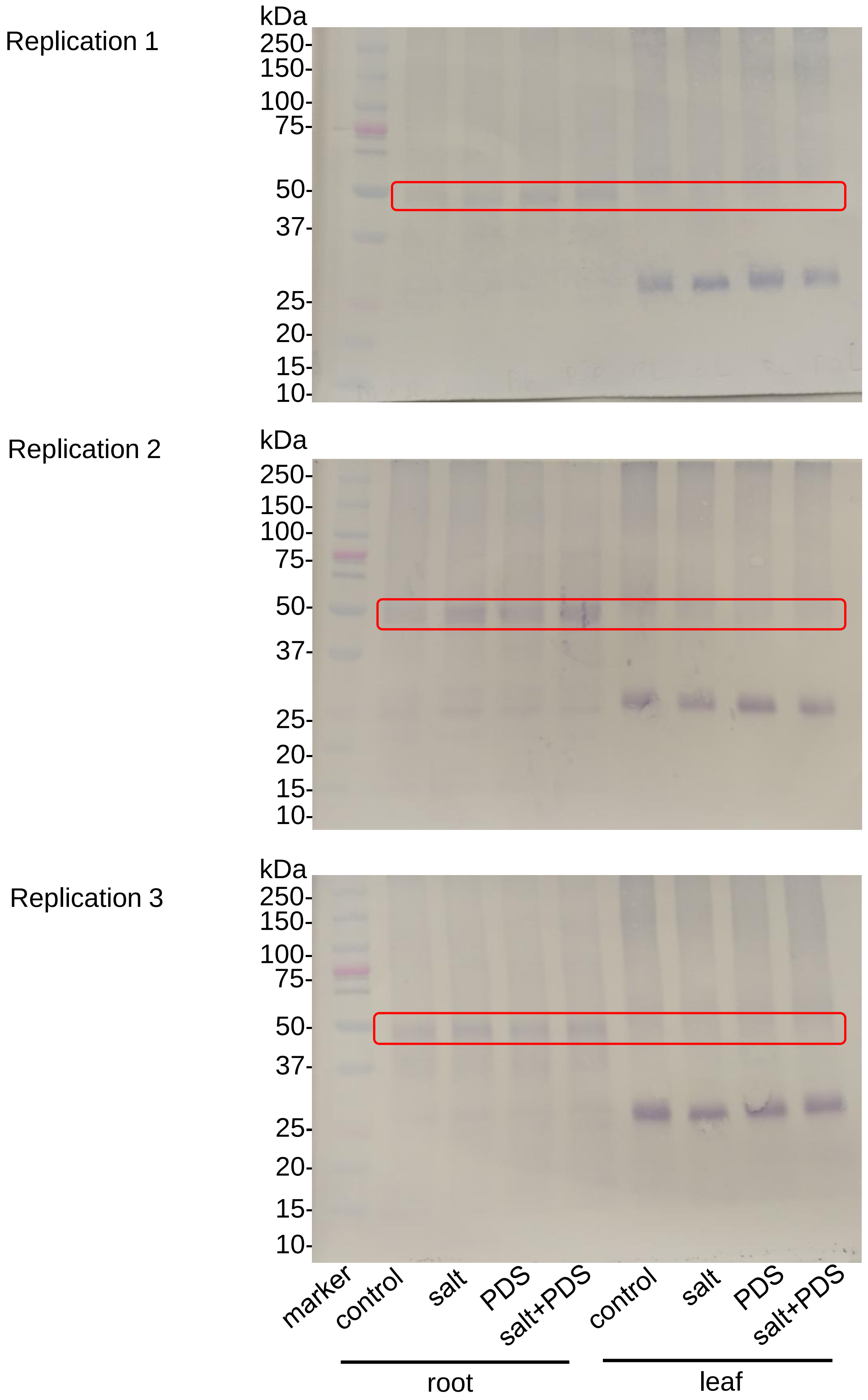

Figure S4. Blots of the entire membrane with anti-histone deacetylase antibody, which were used in Figure 7.
